# Remote Results of 14 Operations of the Shoulder

**Published:** 1918-12

**Authors:** 


**_Remote Results of 14 Operations of the Shoulder._** By M. BERTEIN. *Bulletin de la Société de Chirurgie de Paris.* May 14, 1918.

The author reports briefly a certain number of operations of the shoulder following war injuries. Owing to his experience, he points out the following results :

The most satisfactory intervention in all cases of penetrating fractures by shell-fragments is the total resection of the head of the humerus, with the removal of the splinters. This is unavoidable, when a complete bursting out of the head, or of the neck and head of the bone, has occurred. And even when the bone is fractured lower down, and the head is simply fissured, this must be resected to prevent further infection.

The simple removal of splinters is to be practised only in cases of very limited or non-penetrating fractures.

The incision must be an anterior one. If the aperture due to the projectile is at the back of shoulder, it is of no use for the surgical intervention, and .can only be used for the drainages. However, when the acromion is fractured together with the humerus, the surgeon can make use of a posterior incision. In this particular case, the removal of the acromion leaves a sufficient space for the rugination of the humerus.

A drain is generally placed after the surgical intervention. It is removed after 2 or 3 days. The wound is then washed every two days with peroxyde of hydrogen. No apparatus is used for immobilization.

Following results were obtained : out of 18 cases of injuries of the shoulder, 9 underwent resection of the joint; 5 of them can actually make a good use of their arm; 8 have a swinging arm; death ensued in one case.

The author considers that the mucles of the shoulder must be spared as carefully as possible during the operation. Their action later on may make up, in some degree, for the loss of bones. This sparing of the muscles is obtained by respecting as much as possible the trochanterian insertions. After the intervention, an early suture should be performed to prevent secondary infection. The muscles will be trained progressively by early mobilization.

Later on, secondary myoplastic operations can sometimes lessen the disability of a swinging arm when this could not be avoided.

